# A Peculiar Case of Ligation of the Small Intestines

**Published:** 1892-07

**Authors:** C. A. Cary


### A PECULIAR CASE OF LIGATION OF THE SMALL INTESTINES.

BY C. A. CARY, B. S., D. V. M.

On June 10th I held an autopsy on a fifteen year old mare that had apparently died from enteritis. About 1 P. M. on the 9th I saw the mare after she had been sick five or six hours. Her pulse, temperature, respirations and manifestations of pain indicated that she was affected with acute indigestion; the owner stated that she had been running in a luxurient clover pasture during the previous afternoon and night. Being a visitor at the place and in a hurry, I ordered a purgative of linseed oil; and to relieve the pain, nitrous æther and morphine. About 6 P. M. I saw the mare again; found the pulse 70; the temperature 104° F.; respiration 35; body covered with prespiration; intestinal murmur absent; rectal examination found the rectum and posterior part of the small colon empty, but the mucous membrane of the small colon was covered with an inflammatory mucus exudate; made violent efforts to defecate; abdomen was slightly distended; manifested constant pain, looking around with haggard facial expression at either side with the corresponding hind foot raised from the ground, with the leg extended forward and outward. By the use of morphine and antiseptics the pain and flatulency were controlled to a limited extent. She died at 7 A. M. the next morning. Post mortem examination revealed the fact that the pedecil or stem of a fatty tumor (attached to the mesentary) had become wound around a loop of the small intestines, in such a manner as to completely ligate the intestine at the two points of crossing to form the loop.

In the same mare an aneurysm was discovered in the ileo-cæcal artery, one of the branches of the right fasciculus of the anterior or great mesenteric. There is but little saculation, and only a small portion of the intima was removed; yet a number of mature palisade worms (*Sclerostomum armatum*) were present. The blood current had not been cut off, and as far as I could determine, there were no emboli below the aneurysm. At the origin of the right fasciculus I found a single half grown worm attached to the intima; the intima was perforated only at the point of attachment; and this seems to indicate that the worms do not always depend upon rupture, or tearing of the intima by other causes, for their points of attachment. The mare was raised by the owner, who informed me that she had never been sick previous to the last twenty-four hours of her life.

